# Viewing “p” through the lens of the philosophy of medicine

**DOI:** 10.1186/s13010-019-0077-4

**Published:** 2019-06-11

**Authors:** Sara Asato, James Giordano

**Affiliations:** 1 Department of Physiology, Georgetown University Medical Center, Washington, DC 20057 USA; 2 Departments of Neurology and Biochemistry and Neuroethics Studies Program-Pellegrino Center for Clinical Bioethics, Georgetown University Medical Center, Washington, DC 20057 USA

**Keywords:** Philosophy of medicine, Biostatistics, *P*-value, Research, Research methods, Statistical significance, Ethics

Apropos this journal’s ongoing thematic issue addressing the philosophy of medicine, it is noteworthy to bear in mind philosopher/physician Henk ten Have’s elucidation of any philosophy’s four domains of engagement and application, namely: the metaphysical, the epistemological, the anthropological, and the ethical [1]. While it can be defensibly argued that the practice of medicine entails “art” and is therefore more than mere application of science, it must be also be acknowledged that the sciences most certainly contribute to medicine. The interplay of these scientific and more subtle subjectively intuitive dimensions is well represented by Edmund Pellegrino’s claim that medicine is the “…most scientific of the humanities; and most humane of the sciences” [2]. However, we posit that to accept these humane qualities is not to deny the importance of the scientific – and vice versa - for these merge when engaging tools, knowledge, and skill (i.e.- *tekne*) in pursuit of defined human goods within the clinical encounter.

Eschatological questions, and considerations of existential and transcendent realms of health. Wellness, illness and finitude constituent to medical consideration of the human predicament may involve a number of metaphysical perspectives. But in its scientific character, the metaphysical aspect of (the philosophy of) medicine is, in the main, naturalistic (i.e. - as derived from the methodologic naturalism of the sciences that inform medical research and practice). Given the informational import of the science, the epistemological domain is based upon observation, quantification and experimental manipulation and evaluation of natural substrates and phenomena (i.e. - the bio-psychosocial qualities, conditions, and activities of both patients and clinicians). The anthropological domain obtains that these methods have been, and are developed by humans for application in/for human endeavors, inclusive of the regard and care of human and non-human others (e.g.- human and veterinary medicine). And the goal or perhaps, more aptly, the ends (or *telos*) of this endeavor, a “right and good treatment” of the patient, establishes the ethical domain [3].

To sustain this good, the knowledge used in practice must be current, valid, and relevant to the act(s) of medicine. In this way, the quest for and use of scientific knowledge (from the physical, natural, life and social sciences) is intrinsic to the philosophical domains and real acts of medicine [4]. This quest necessitates, and is reliant upon accepted standards and conduct of research enterprises. Appreciating that science involves the use of ever more capable tools to develop and fortify theories that are used in practice [5], it then follows that any and all research in the disciplines constituent to medicine must utilize methods that are contemporary.

Recently, there has been – and continues to be – discussion, if not debate, about the relative value of *p*-values < 0.05 [6]. To date, p-values have been used to determine thresholds for statistical significance. However, studies of the significance of *p*-values are suggesting limited value of its intended use [7–9]. However, eliminating the use of *p*-values altogether may be just as problematic. Although experiments certainly had been conducted, and results evaluated prior to the *p*-value being introduced in the 1920s [10], it now seems inapt to conduct scientific research without some sort of inferential statistics. To wit, one proposed solution was that researchers should justify their use of specific *p*-values, rather than arbitrarily employ *p* < 0.05 [11].

Yet, using *p*-values that are more stringent than the 0.05 threshold (e.g. 0.01, 0.005, 0.001) may also be problematic. Researchers may use false reporting, fishing, cherry-picking of subjects, selective reporting, muddled thinking, or “rubber stamping” in order to attain a “more rigorous” *p*-value (i.e.-, what is colloquially referred to as “p-hacking”) [12]. Thus, it becomes important to query if and to what extent adoption of more stringent *p*-values could have on the viability, validity and value of research outcomes.

Conversely, a shift to the use of more stringent *p*-values could obtain benefit(s) in and to particular domains of biomedical research (e.g. - both “low-tech”, such as studies of complementary medical approaches; and “high-tech”, such as investigations of emerging biotechnologies and techniques) in which sample size, and magnitude of effect(s) might be limited. If such studies were held to a higher standard, the legitimacy and worth of findings – despite inherent limitations – might increase, and the translational utility of such outcomes become more widely accepted. This offers possibility for continuity of extant support, and generation of additional funding for further research.

The use of more rigorous metrics, validation of studies (that were heretofore limited by sample size, etc.) and amenability toward expedited translation of research findings to practice might also foster increasing insurance support for novel and emerging methods and technologies. This might enable both macro- and micro-economically feasible use of resources, and permit more (affordable) options for a range of low-to-high tech health promotion and care services [13]; which could be especially helpful in medically underserved areas (in developed, developing, and non-developed nations) [14].

Of course, this would incur some consideration of the validity and value of prior studies (with statistical significance established at *p* < 0.05). Is the relative worth (if not integrity) of these studies’ findings void? Should findings of these previous studies be re-evaluated using more stringent *p*-values? Should these new results determine which studies require reinterpretation of findings, revision of method, or outright rejection of outcomes and conclusions? Or, should the debate about *p*-values be seen as a “sign of the times”, and represent a coming of age that demands greater granularity in statistical methods used given the range of techniques and technologies employed or under examination?

Indeed, Ronald Fisher’s introduction of the *p*-value in the 1920s was more for determining if the probability of outcomes would warrant evaluation and/or replication [15]. We believe that there is (still) merit to Fisher’s view and intent. *P*-values can – and we assert, should - be seen as a threshold for either (1) the relative acceptability of research findings, or (2) prompting of further examination, assessment, and validation. Thinking logically, *p*-values were created by humans to test for chance happenings, which are, at least in part, produced by human error (and error in the use or function of tools and techniques created by humans). The research community may be facing an opportunity to broadly acknowledge the explicit obligation of science to be self-critical and self-revising [10], and through such a lens hold a mirror to itself and to medicine in examination of the ways that research outcomes are evaluated, regarded and used. We value that most surely as significant.
